# A novel molecular mechanism for a long non-coding RNA PCAT92 implicated in prostate cancer

**DOI:** 10.18632/oncotarget.25940

**Published:** 2018-08-21

**Authors:** Pushpinder Singh Bawa, Samathmika Ravi, Swagatika Paul, Bibha Chaudhary, Subhashini Srinivasan

**Affiliations:** ^1^ Institute of Bioinformatics and Applied Biotechnology, Biotech Park, Electronic City Phase I, Bangalore, India; ^2^ Manipal University, Manipal, Karnataka, India

**Keywords:** lncRNA, PCAT92, prostate cancer, ABCC4, ZIC2

## Abstract

The role of many lncRNAs in cancer remains elusive including that for a Prostate Cancer Associated Transcript 92 (PCAT92). PCAT92 shares the locus on chromosome 13 with ABCC4 gene, known to be implicated in prostate cancer. It has been shown that PCAT92 and ABCC4 are up-regulated in prostate cancer samples from multiple transcriptome datasets. Among the prostate cancer cell-lines LNCaP showed maximum overexpression of PCAT92 compared to control cell-line RWPE-1. We have shown that knockdown of PCAT92 in LNCaP cells reduces cell viability and proliferation and down-regulates ABCC4 transcript/protein expression. The shared region between PCAT92 and ABCC4 has a binding site for an oncogenic transcription factor (ZIC2) which is also upregulated in the majority of datasets studied here. ZIC2 binding to the predicted ABCC4 promoter has been confirmed using pull-down assay. Interestingly, under PCAT92 knockdown condition, there is a reduction in the ZIC2 binding to ABCC4 promoter indicating the potential involvement of PCAT92 in the recruitment of ZIC2. We have identified distinct regions on PCAT92 with potential to bind to ZIC2 non-DNA binding Zinc-finger domain and potential for triplex formation near ABCC4 promoter region, which have been experimentally validated. Together, these observations and localization in the nucleus suggests that PCAT92 may play a role in prostate cancer by increasing the local concentration of ZIC2 by forming RNA-DNA triplex near ABCC4 promoter thus helping in recruitment of ZIC2 for ABCC4 regulation.

## INTRODUCTION

Long non coding RNAs (lncRNAs) are bringing a new level of understanding of cancer biology. Using high-throughput sequencing, a large number of cancer-associated lncRNAs are being discovered and involvement of many of them in cancer is slowly emerging. The mechanism of MALAT1, one of the earliest lncRNAs associated with cancer, is still emerging. Using mouse knockout models, it was shown that a set of metastasis associated genes were deregulated, strongly indicating its basis as a regulator of gene expression [1]. Among other lncRNAs, ANRIL, encoded in 9p21, a hotspot region for polymorphisms associated with cancer, predisposes to a variety of cancer types including breast, ovarian and prostate [2–4]. Overexpression of HOTAIR correlates with colorectal carcinogenesis and poor prognosis [5]. Knockdown studies of HOTAIR in CRC has shown its potential role in cell proliferation, migration, invasion, apoptosis and radiosensitivity [5]. A few lncRNAs have been reported as tumor suppressors including GAS5 [6] and MEG3 [7]. The expression of these lncRNAs was shown to be elevated in normal prostate epithelial cells but reduced in prostate cancer cell lines.

More recently, using high-throughput transcriptome sequencing, it has been shown that almost a quarter of abundant transcripts in prostate cancer are lncRNAs with many that are still unannotated [8]. This is interesting considering that the first lncRNA that made its way into clinical practice was PCA3 for the early detection of prostate cancer from urine samples [9]. Currently, efforts to translate several other prostate cancer associated lncRNAs (PCATs) are in progress. PCAT1, a transcript from the 8q24 MYC prostate locus, has been characterized functionally [8]. PCAT1 acts both as a candidate for transcriptional repression by PRC2 as well as activator of cell proliferation through transcriptional regulation of target genes such as MYC. PCAT14 has been shown to distinguish benign and malignant cancer subtypes and the loss is linked with disease aggressiveness and recurrence [10]. It has been demonstrated that PCAT29 has a cancer suppressive phenotype using cell proliferation, migration, tumor growth and metastasis [11]. The involvement of PCAT6 in proliferation and invasion of lung cancer has been shown using knockdown experiments [12]. PCAT2, which is part of the 8q24 risk locus harboring variants associated with prosate cancer, predict susceptibility to prostate cancer in men of African Ancestry [13].

One of more established mechanism of action of lncRNAs is by regulation of genes within the same locus. For example, PCA3, located in intron 6 of the PRUNE2 gene, is co-expressed along with PRUNE2 in the nucleus to form a double-stranded RNA [14]. The lncRNA CTBP1-AS is located in the antisense strand of CTBP1 [15, 16]. ANRASSF1, an antisense RNA of RASSF1A gene, is another example of a lncRNA that suppresses the transcription of a neighboring gene [17]. lincRNA-p21 predicted to be associated with the p53 is found to be directly regulated by p53. It forms a lncRNA-RNP with a nuclear factor and serves as a global transcriptional repressor facilitating p53- mediated apoptosis [18]. Another hallmark example is HOTAIR, which is encoded in the HOXC cluster and is a strong predictor of breast cancer metastasis. A comparative breast cancer study of profiling lncRNA have shown that expression of HOTAIR is sufficient to drive breast cancer metastasis [19].

We have previously shown that PCAT92 is located closely to ABCC4 gene on chromosome 13 [20]. Expression of ABCC4 is significantly higher in prostate cancer cells as compared to adjacent benign cells [21] suggesting that the gene is either merely linked with cancer or that the gene is somehow involved with the formation and progression of cancer. It has been determined that the expression of ABCC4 is positively correlated with androgen levels in prostate cancer patients. Addition of androgens in prostate cancer cells significantly upregulated ABCC4 and the addition of anti-androgens decreased expression [21]. These results warrant further study of the ABCC4 gene and its association with androgen levels as potential therapeutic targets in prostate cancer.

Zinc-finger transcriptional regulators ZIC1 through ZIC5 have been associated with increase in transcriptional activity of many genes [22]. ZIC2 is one of member of ZIC2 gene family have been shown to have oncogenic properties [23]. In pancreatic ductal adenocarcinoma ZIC2 knockdown induced cell apoptosis and ZIC2 over-expression enhanced the cellular proliferation [24]. ZIC2 overexpression has been reported in epithelial ovarian tumors samples and is correlated strongly with the clinical course of cancer [25]. Increase in expression of ZIC2 has also been reported in hepatocellular carcinoma. It has been shown to promote tumor growth and metastasis by directly binding to the PAK4 promoter and modulating its activity [26]. In prostate cancer it has been shown that ZIC2 has functional significance at the molecular and cellular levels in the initiation of prostate adenocarcinoma (PRAD) and the progression to metastatic and/or castration resistant prostate cancer (CRPC) [27].

Here our work demonstrates how PCAT92 transcript is regulating the expression of ABCC4 by recruiting ZIC2 at the ABCC4 promoter region and forming triple helix near the promoter site to stabilize the binding of ZIC2 at ABCC4 promoter region.

## RESULTS

### Organization of PCAT92/ABCC4 locus

**Figure 1:**
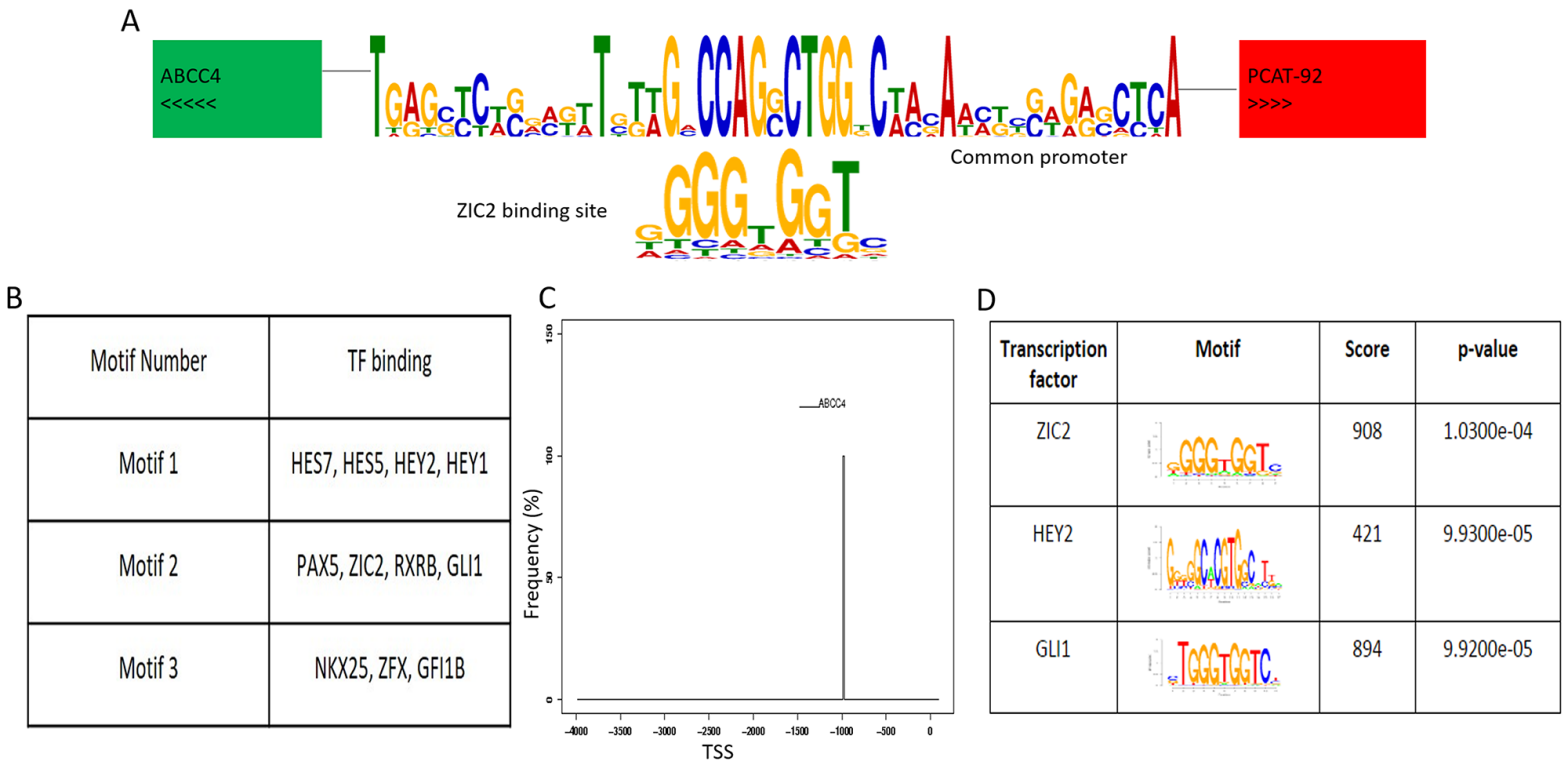
ABCC4-PCAT92 locus **(A)** This image represents the divergent chromosomal location of ABCC4 and PCAT92 on chromosome 13. It also shows the potential transcription factor, ZIC2 binding site at the 4kb shared region between ABCC4 and PCAT92. **(B)** The top three motif predicted using MEME package at the 4kb shared region between ABCC4 and PCAT92. List of transcription factors having the binding site at these motif searched using TOMTOM. **(C)** The predicted binding site of ZIC2 at the shared 5’UTR region. **(D)** List of transcription factor having the highest significant score to bind the shared locus along with their binding domain.

**Figure 2:**
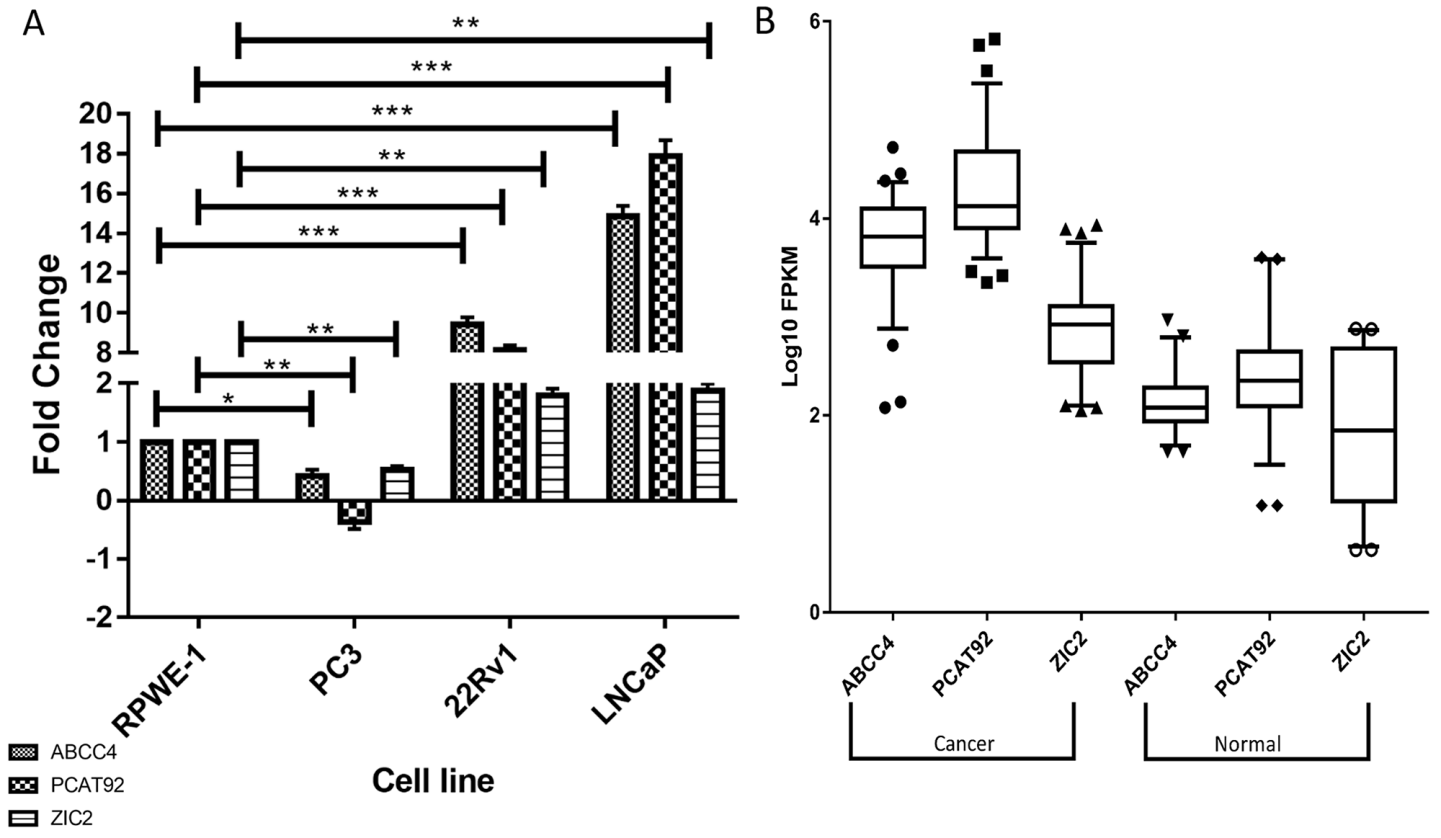
Expression profile of ABCC4, PCAT92 and ZIC2 in prostate cancer **(A)** Expression status of ABCC4, PCAT92 and ZIC2 in three prostate cancer cell lines (PC3, 22Rv1 and LNCaP) in comparison with prostate normal (RPWE-1). It can been seen that all the three genes showed a higher expression status in LNCaP and 22Rv1 versus prostate normal. Actin was used as a housekeeping gene to calculate ΔΔCt. **(B)** Box whiskers plot plotted using RNA-Seq data analysis showing higher expression of ABCC4, PCAT92 and ZIC2 in 78 prostate cancer patient samples when compared to adjacent benign tissues. (^***^ P<=0.001, ^**^ P<=0.01 and ns P>0.05).

PCAT92 and ABCC4 are only 4305 bases apart on chromosome 13 in a divergent configuration sharing the 5’ regulatory region (Figure 1A). Promoter prediction methods found binding sites for eleven transcription factors, GFI1B, HEY2, ZXRB, ZIC2, HEY1, ZYF, NKX2-5, PAX5, GLI1, HES5 and HES7 within the shared loci (Figure 1B). Out of these not only was ZIC2 among the top three transcription factor based on significance (Figure 1D) but is also significantly overexpressed in prostate cancer tissues and cell lines like PCAT92 and ABCC4 (Figure 2A and 2B). The ZIC2 consensus was found on the negative strand between -987 and -972 from ABCC4 transcription start site (Figure 1C). Interestingly, ZIC2 locus is also in chromosome 13 a mere 5 million bases away from ABCC4 locus.

### Expression status of PCAT92, ABCC4 and ZIC2 in cancer

It was reported that PCAT92, ABCC4 and ZIC2 are overexpressed in multiple prostate cancer datasets in the public repository (SRP002628 and ERP000550) [20]. The over-expression of these three genes are here validated in two prostate cancer cell lines LNCaP and 22Rv1 and is compared to their expression in prostate normal cell line RPWE-1 (Figure 2A). Another prostate cancer cell line, PC3 showed the least expression of ABCC4, PCAT92 and ZIC2. ABCC4 has previously been reported to be down-regulated in PC3 [28]. One of the major reason for the lower expression of ABCC4 and PCAT92 in PC3 cell line may be the androgen independent nature of the cell line. The expression status of the three transcriptsare further validated in a publicly available transcriptome data [SRP005908] consisting of 78 prostate cancer patient samples and 38 benign prostate tissue sample. We performed differential gene expression analysis to calculate the fold change in the expression of these transcripts in prostate cancer sample with respect to benign prostate tissue. The box-whiskers plot in Figure 2B shows cancer-specific overexpression of ABCC4, PCAT92 and ZIC2 in 78 other prostate cancer samples relative to 38 adjacent benign tissues. The Pearson correlation coefficient between PCAT92 and ABCC4 expression profile across the 78 samples is 0.843 when compared to 0.148 between PCAT92 and housekeeping gene. No correlation is expected for ZIC2 with ABCC4 and PCAT92. However, the moderate level of correlation coefficient of 0.586 between PCAT92 and ZIC2 may be from shear over expression in cancer.

### Knockdown studies of PCAT92 and ABCC4

**Figure 3:**
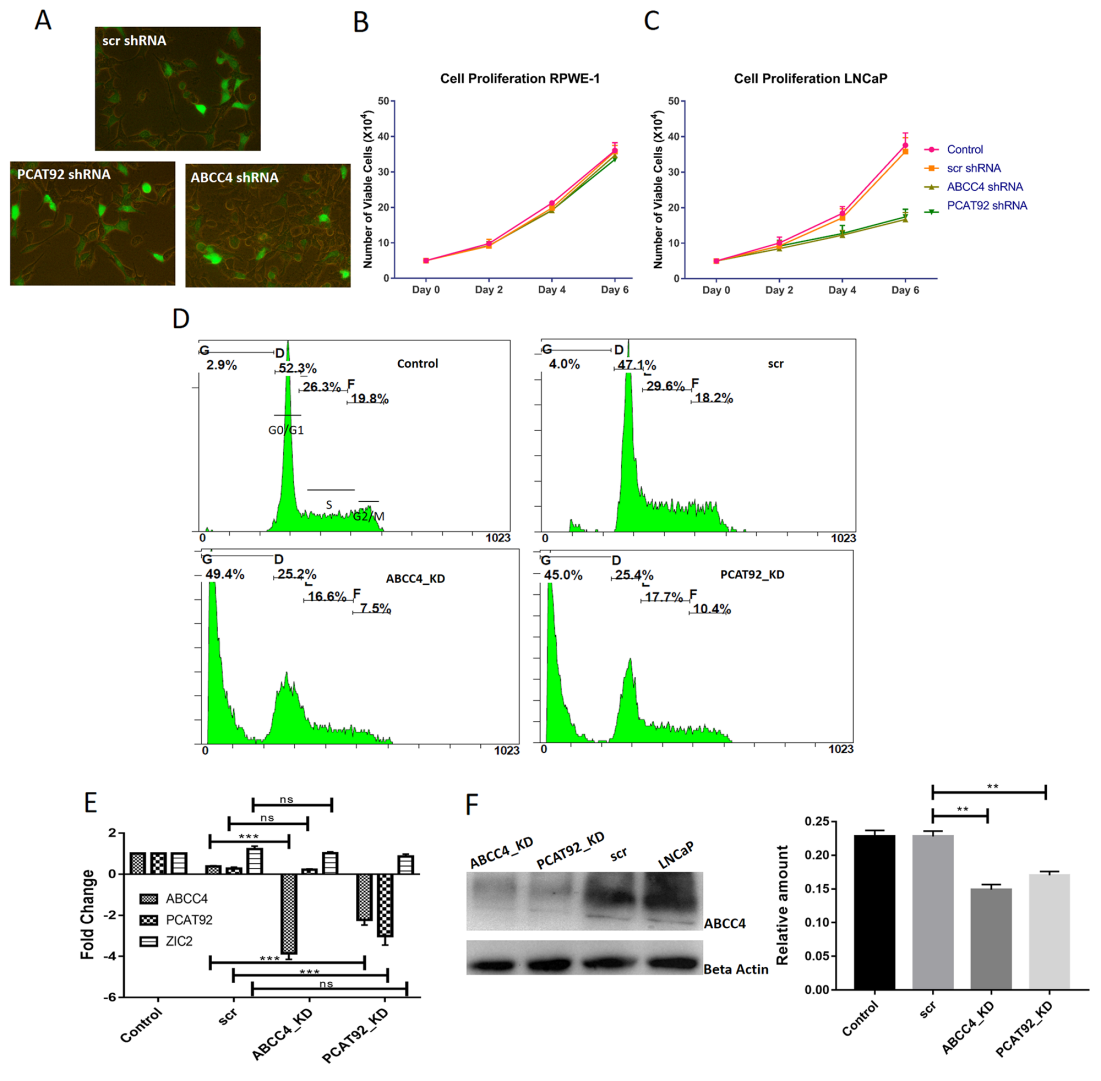
Proliferation assay in LNCap cells using PCAT92 and ABCC4 knockdown experiments **(A)** LNCaP cells transfected with shRNA constructs against ABCC4, PCAT92 and scrambled. Based on the number of fluorescent cells, it was observed that transfection efficiency was about 75%. **(B)** MTT assay showing the effect of ABCC4 and PCAT92 knockdown on RPWE-1 cell viability. As we have shown that RPWE-1 cells does not express ABCC4 and PCAT92, we observed that there no reduction in cell viability under both ABCC4 and PCAT92 knockdown when compared to control indicating that there is no off-target effect due to shRNA. **(C)** MTT assay showing the effect of ABCC4 and PCAT92 knockdown on LNCaP cell viability. It was observed that there a reduction in cell viability under both ABCC4 and PCAT92 knockdown when compared to control. This indicates that both ABCC4 and PCAT92 are required for cancer cell growth. **(D)** FACS analysis showing an increase in number of dead cell population under the knockdown condition indicating that ABCC4 and PCAT92 has a role in cell proliferation. No cell arrest and any specific cycle stage was observed. **(E)** qRT-PCR results from ABCC4, PCAT92 and ZIC2 post knockdown of ABCC4 and PCAT92: A significant reduction in the transcript level of ABCC4 and PCAT92 was observed in their respective knockdown LnCAP cells. Interestingly, upon PCAT92 knockdown, a significant reduction in the transcript level of ABCC4 was also observed, indicating a possible regulatory role of PCAT92 in ABCC4 gene regulation. No significant change in the PCAT92 transcript level was observed upon ABCC4 knockdown. No significant change in the level of ZIC2 was observed under both ABCC4 and PCAT92 knockdown condition. Actin was used as a housekeeping gene to calculate ΔΔCt. Both the knockdowns were performed in three independent batches. **(F)** Western blot analysis, showing a significant reduction in ABCC4 protein level in both ABCC4 and PCAT92 knockdown condition, thereby strengthening our prediction of PCAT92 playing a role in ABCC4 gene regulation. The bars in the graph are plotted based on the band intensity observed in the western blot. Actin was used as a control. (^***^ P<=0.001, ^**^ P<=0.01 and ns P>0.05).

Figure 3 summarizes results from knockdown of ABCC4 and PCAT92 transcript in LNCaP cell line using shRNA. We used three constructs targeting different regions against both ABCC4 and PCAT92 RNA for efficient knockdown. Scrambled shRNA (scr shRNA) was used as a control. All the constructs were tagged with GFP. Figure 3A shows the efficiency of transfection of constructs into LNCaP cells. We used lentiviral vector system for transfection. MTT assay was performed to understand the effect of ABCC4 and PCAT92 knockdown on cell viability. The knockdown cells were grown from Day 0 till Day 6 and MTT assay was performed after every 48 hours. Figure 3C shows a reduction in cell viability upon ABCC4 and PCAT92 knockdown in LNCaP cells independently. Figure 2A shows that RPWE-1 expresses the least amount of ABCC4 and PCAT92, which is used this as a control for MTT assay. Figure 3B shows no change in cell viability from knockdown of both ABCC4 and PCAT92 in RPWE-1 cells. This shows that the reduction in cell viability observed in LNCaP cells is due to knockdown of ABCC4 and PCAT92 transcripts and there is no off-target effect. As the maximum effect on cell viability was seen on Day 6, we performed FACS analysis on these cells to study the effect of knockdown on cancer cell proliferation. Under both knockdown conditions we observed ∼45% increase in the number of dead cell population as compared to scr (scrambled shRNA) without any significant arrest at any specific cell cycle phase (Figure 3D). The similar effect on cell viability and cell proliferation under both ABCC4 and PCAT92 knockdown condition suggests that both genes are involved in same pathway in prostate cancer. Significant knockdown of ABCC4 (2.5 fold at RNA levels and 1.58 fold at protein level) did not have any effect on the PCAT92 and ZIC2 RNA levels. However, 2.5 fold knockdown of PCAT92 transcript resulted in significant reduction of ABCC4 at both RNA (2 fold) and protein (1.36 fold) levels (Figure 3D, 3E). PCAT92 knockdown also has no significant effect on ZIC2 RNA level (Figure 3D). This would suggest that PCAT92 is upstream to ABCC4 expression. To check for the change in gene expression upon knockdown, transcriptome sequencing was performed on shRNA knockdown ABCC4 and PCAT92 LNCaP cells. Differential gene expression profile of both knockdown compared to the control is also concordant. Very small number of genes were up-regulated in both knockdown conditions. A significant (80%) overlap was observed in the genes which are down-regulated under the two knockdown conditions. Pathway analysis of both the knockdown showed common pathway PI3K-Akt, which is a very well-known cell proliferation pathway. This suggests that PCAT92 and ABCC4 might be involved in regulation of the same pathway. Interestingly, there are reports of cross talk between AR pathway and PI3K-Akt pathway [29, 30].

### Binding of ZIC2 to ABCC4 promoter

**Figure 4:**
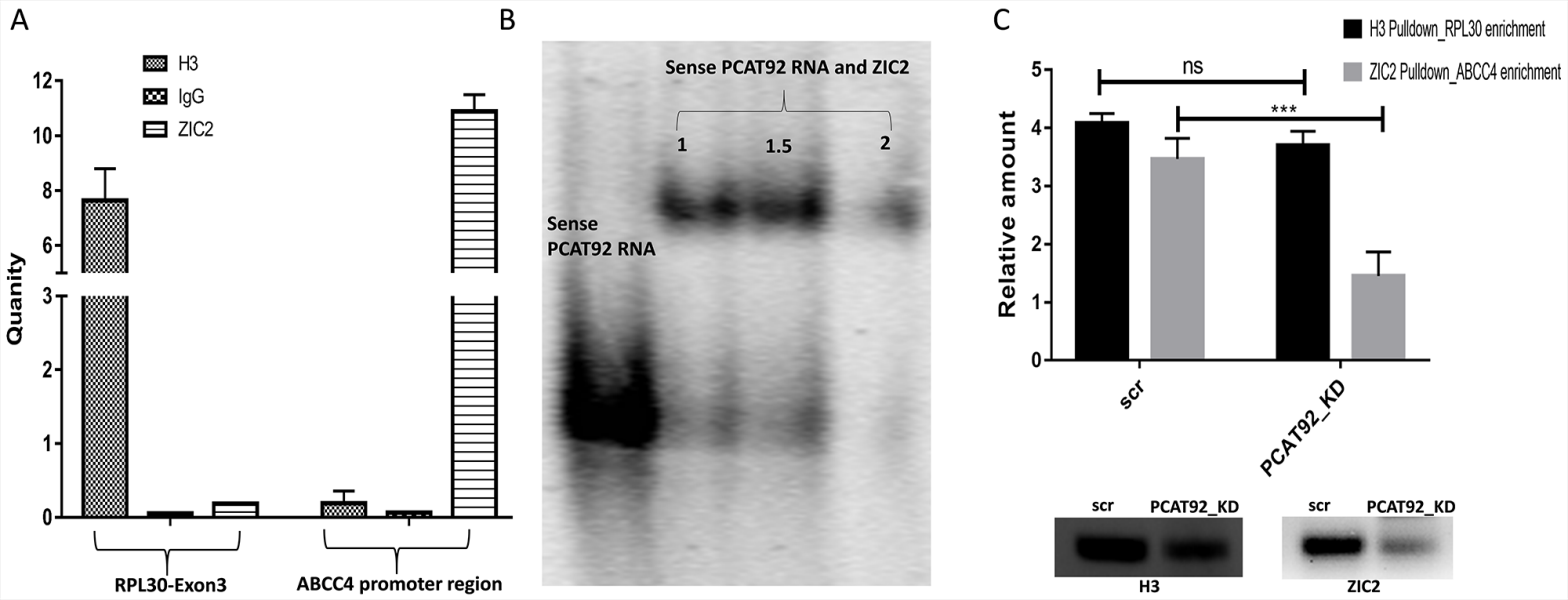
Pulldown/binding assays for ZIC2-ABCC4 **(A)** ChIP RTPCR to show the enrichment of ABCC4 promoter region using ZIC2 pulldown. H3 pulldown for enrichment of RPL30 exon 3 was used as a positive control and anti-rabbit IgG was used as a negative control. Three independent set of experiment was performed. **(B)** Mobility shift assay, confirming the binding of ZIC2 at ABCC4 promoter region. **(C)** ChIP-PCR under PCAT92 knockdown a reduction in the enrichment of ABCC4 promoter region was observed indicating a possible interaction between PCAT92 and ZIC2 to help the later in forming a stable complex at the promoter region. H3 pulldown for enrichment of RPL30 exon 3 was used as a control. The bars in the graph are plotted based on the band intensity observed in the immunoblot gel. Three independent set of experiment was performed. (^***^ P<=0.001, ^**^ P<=0.01 and ns P>0.05).

Binding affinity of ZIC2 at the ABCC4 promoter region in cell line is studied using ChIP-RTPCR with anti-ZIC2 antibody. Primers flanking the predicted ZIC2 binding site on ABCC4 promoter region was made. As shown in Figure 4A, enrichment of the ZIC2 binding site on ABCC4 promoter region is observed using anti-ZIC2 antibody. Enrichment of RPL30 exon 3 using anti-histone H3 antibody was used as positive control and rabbit IgG antibody was used as a negative control. Oligos’ of 58 bp flanking the ZIC2 binding motif on the ABCC4 promoter was synthesized and then annealed to make a dsDNA. The annealed oligo was 5’end labelled with [γ-32P] ATP. A region of ZIC2 containing the five zinc finger domains was cloned, expressed and purified. Figure 4B shows the result of electrophoretic mobility shift assay between the synthesized dsDNA of ABCC4 promoter and purified zinc finger domains of ZIC2. A clear shift in the gel band in EMSA and ChIP-RTPCR together confirms the binding of ZIC2 at the ABCC4 promoter region. On the other hand, under PCAT92 knockdown condition ChIP-PCR showed significantly reduced enrichment of ABCC4 without any significant change in the enrichment of RPL30 exon 3 (Figure 4C). This suggests that PCAT92 may directly/indirectly be implicated in ZIC2 binding to ABCC4 promoter region.

### Interaction studies between PCAT92 and ZIC2

**Figure 5:**
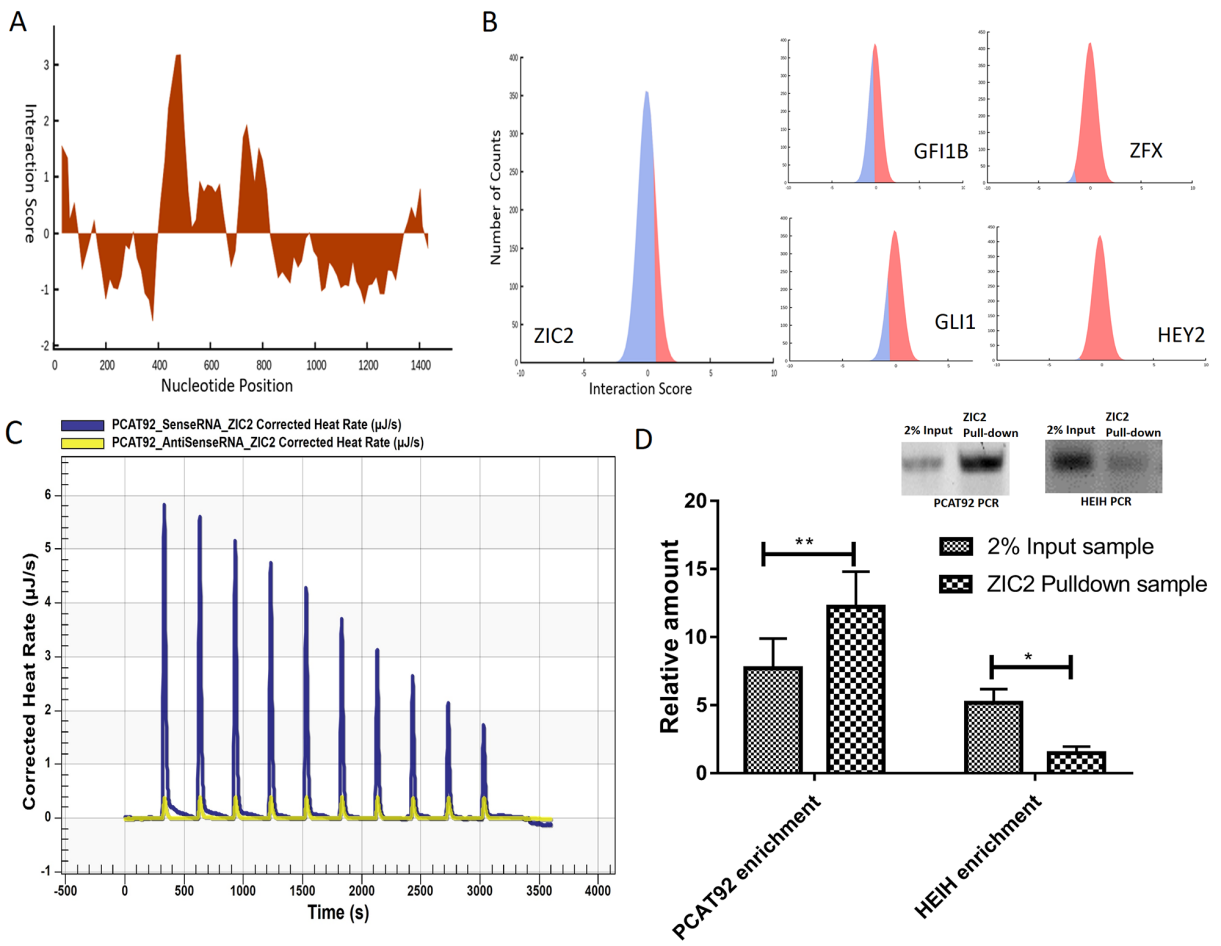
Interaction studies between PCAT92 and ZIC2 **(A)** Shows the interacting strength between of PCAT92 and ZIC2 and the region of PCAT92 which has the maximum binding potential with ZIC2. **(B)** Graph shows interaction strength between the non-DNA binding domain of ZIC2 and PCAT92. A 72% interaction strength was observed between the two domains compared to GFI1B (43%), GLI1 (39%), ZFX (8%) and HEY2 (1%). **(C)** NanoITC result showed a sequencing specific interaction between PCAT92 transcript and ZIC2. Blue indicate the interaction between sense PCAT92 with ZIC2 and yellow indicate antisense PCAT92 RNA with ZIC2. **(D)** RNA immunoprecipitation results validating interaction between PCAT92 RNA and ZIC2 protein *in vivo*. As a positive control for RNA-protein crosslinking we amplified HEIH RNA. Amplification was observed in both PCAT92 and HEIH RNA in 2% aliquot sample, where as in ZIC2 pulldown sample amplification was observed only in PCAT92 RNA but not in HEIH RNA indicating interaction between PCAT92 and ZIC2. Three independent set of experiment was performed. (^***^ P<=0.001, ^**^ P<=0.01 and ns P>0.05).

One possible mechanism by which PCAT92 may affect binding of ZIC2 to ABCC4 promoter is by directly binding to ZIC2 to stabilize the complex. Prediction of the binding site between ZIC2 and PCAT92 using catRAPID revealed that ZIC2 may bind to PCAT92 via a non-DNA binding zinc finger domain with maximum interaction propensity in the region 301-352 aa (UniProtKB-O95409) and PCAT92 region between exon2-exon3 (443-504 bp) as shown in Figure 5A. This interaction strength (72%) was significantly higher compared to the ability of PCAT92 to bind to zinc finger domains of other transcription factors (Figure 5B). The predicted region of PCAT92 was cloned and then transcribed *in vitro*. The transcribed RNA was then titrated onto the purified zinc finger domains of ZIC2 using Nano Isothermal Titration Calorimetry (NanoITC). This validated the interaction between the predicted PCAT92 RNA strand and the zinc finger domains of ZIC2. Antisense strand to the predicted PCAT92 RNA region was also cloned and transcribed to be used as negative control. As shown in Figure 5C there is reduced interaction between the equimolar antisense RNA to the zinc finger domains of ZIC2 as compared to sense RNA. This suggests a possible sequence specific interaction between PCAT92 and ZIC2 and role of PCAT92 in recruiting ZIC2 to the promoter site. The thermodynamic parameters elucidated for the binding of the RNA to the DNA are collated in Table 1. A threefold difference in the association constant was observed between sense PCAT92 RNA and ZIC2 protein, when compared to antisense PCAT92 RNA and ZIC2 protein. For *in-vivo* validation of interaction between PCAT92 and ZIC2, we performed RNA Immunoprecipitation (RIP). The cells were fixed and RNA and protein were crosslinked. Using anti-ZIC2 antibody RNA-protein complexes were pulled down. RNA-Protein complex were broken by reverse crosslinking and the resulting RNA was subjected to cDNA synthesis. HEIH, one of the well-established non-coding RNA [31] known to bind to protein, was used as positive control. As seen in the Figure 5D significant enrichment in PCAT92 was observed using pull down by anti ZIC2 antibody, indicating an interaction between PCAT92 RNA and ZIC2 protein. Figure 5D also shows the lack of enrichment of HEIH RNA, indicating a specific interaction between PCAT92 RNA and ZIC2 protein.

**Table 1:** Thermodynamics parameters of PCAT92-ZIC2 interaction and ABCC4-PCAT92 interaction observed using NanoITC

Parameter	Antisense PCAT92 RNA - ZIC2 Protein	Sense PCAT92 RNA - ZIC2 Protein	Random RNA- ABCC4 DNA	PCAT92 RNA-ABCC4 DNA
**Ka M**^-^	1.000E6	1.000E9	3.516E6	1.655E7
**-TΔS kJ/mol**	52.40	89.58	-150.1	100.1
**ΔG kJ/mol**	-34.25	-51.37	-37.36	-41.21
**ΔS J/mol**^-^**K**	-175.7	-300.5	503.6	-335.7

### Triplex forming capability of PCAT92 near ABCC4 promoter region

**Figure 6:**
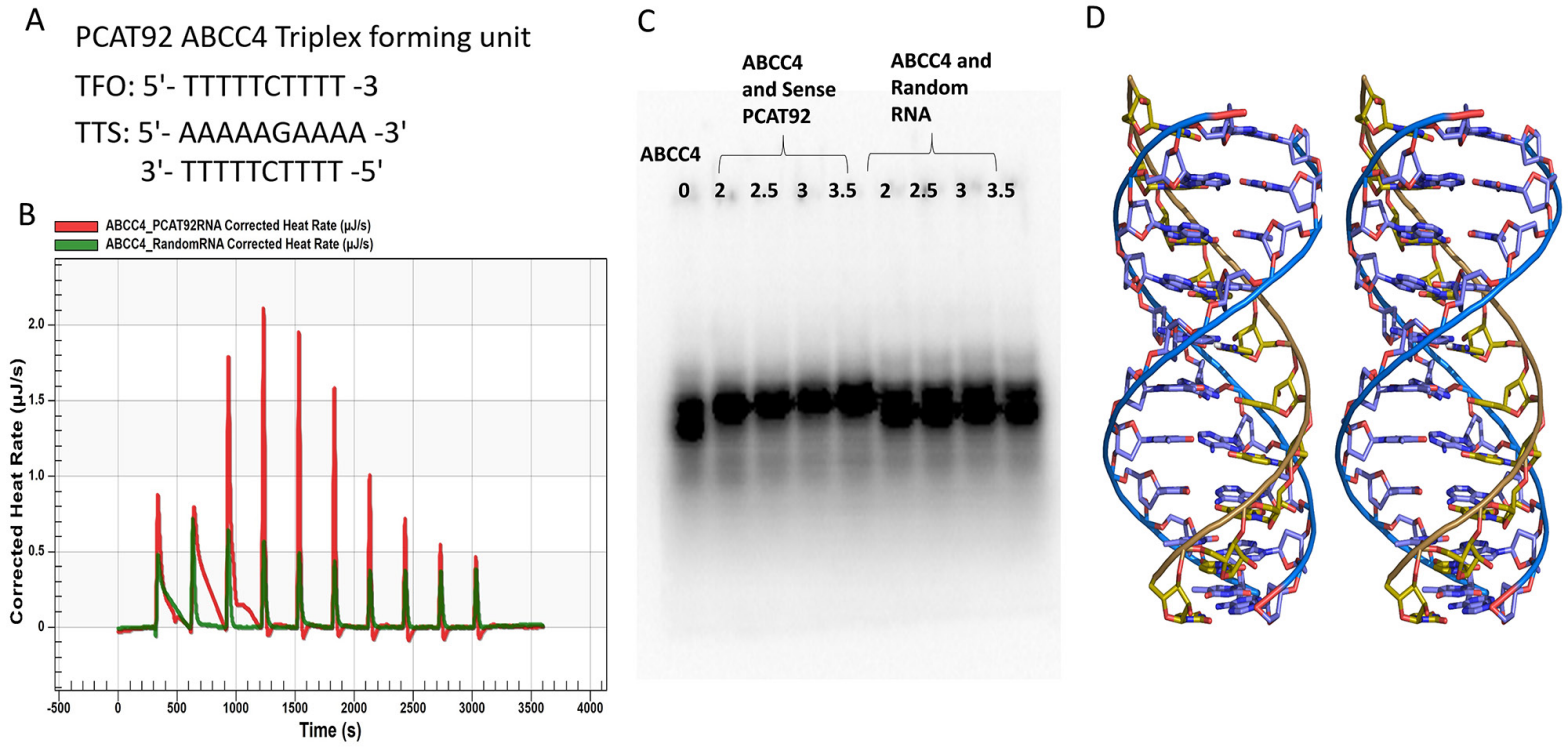
PCAT92-ABCC4 Triplex **(A)** Triplex forming oligo (TFO) on PCAT92 strand and Triplex Target Site (TTS) on ABCC4. **(B)** NanoITC result showed a sequencing specific interaction between PCAT92 transcript and ABCC4. Red indicate the interaction between sense PCAT92 with ABCC4 and green indicate random RNA with ABCC4. **(C)** EMSA showing a sequence specific interaction between ABCC4 and sense PCAT92 RNA forming triplex. Random RNA was used as a negative control. **(D)** The model of the predicted PCAT92-ABCC4 Triplex.

Above observations require the maintenance of the local concentration of PCAT92 for the recruitment of ZIC2 at ABCC4 promoter site. Since lncRNAs are known to have the ability to form triple helical structure with chromosomal DNA [32], the ability of PCAT92 binding to the chromosomal DNA near the ABCC4 promoter site was explored. Using Triplexator, a highly significant triplex forming oligo (TFO) with zero mismatches was predicted for PCAT92 transcript having triplex forming potential near ABCC4 promoter region. As a negative control for this prediction, we used same criteria for other lncRNAs, like XIST and HOTAIR, no significant TFO were predicted. Figure 6A shows the predicted DNA and the RNA sequence with propensity to form RNA-dsDNA triplex. We have built a model using the B-DNA configuration for chromosomal DNA with the most significant RNA strand based on Hoogsteen hydrogen bonding in the major groove (Figure 6D). Thermodynamic characterization of the ABCC4 DNA and PCAT92 RNA was performed using Nano Isothermal Titration Calorimetry (NanoITC). The calorimetric profiles for the binding of both PCAT92 RNA and a random RNA to the ABCC4 DNA are presented in Figure 6B. Binding in both the case was characterized by the presence of exothermic peaks that followed each injection of the RNA to the DNA. The thermodynamic parameters elucidated for the binding of the RNA to the DNA are collated in Table 1. The ITC data for the binding of random RNA to the ABCC4 DNA yielded an association constant of 3.516E6 M^-^. The binding of PCAT92 RNA to ABCC4 DNA yielded a higher association constant of 1.655E7 M^-^ suggesting a possible sequence specific interaction between PCAT92 RNA and ABCC4 DNA. The Gibbs energy change (ΔG) for random RNA was found to be higher than PCAT92 RNA for ABCC4 DNA showing the spontaneity of the interaction is more for PCAT92 RNA than random RNA. Figure 6C is the EMSA results confirming the triplex formation between the predicted region of ABCC4 DNA and PCAT92 RNA. A dosage dependent interaction between ABCC4 DNA and PCAT92 RNA was observed. A random RNA of similar length showed no dosage dependent interaction with ABCC4.

### PCAT92 co-localizes with ZIC2 in the cell

**Figure 7:**
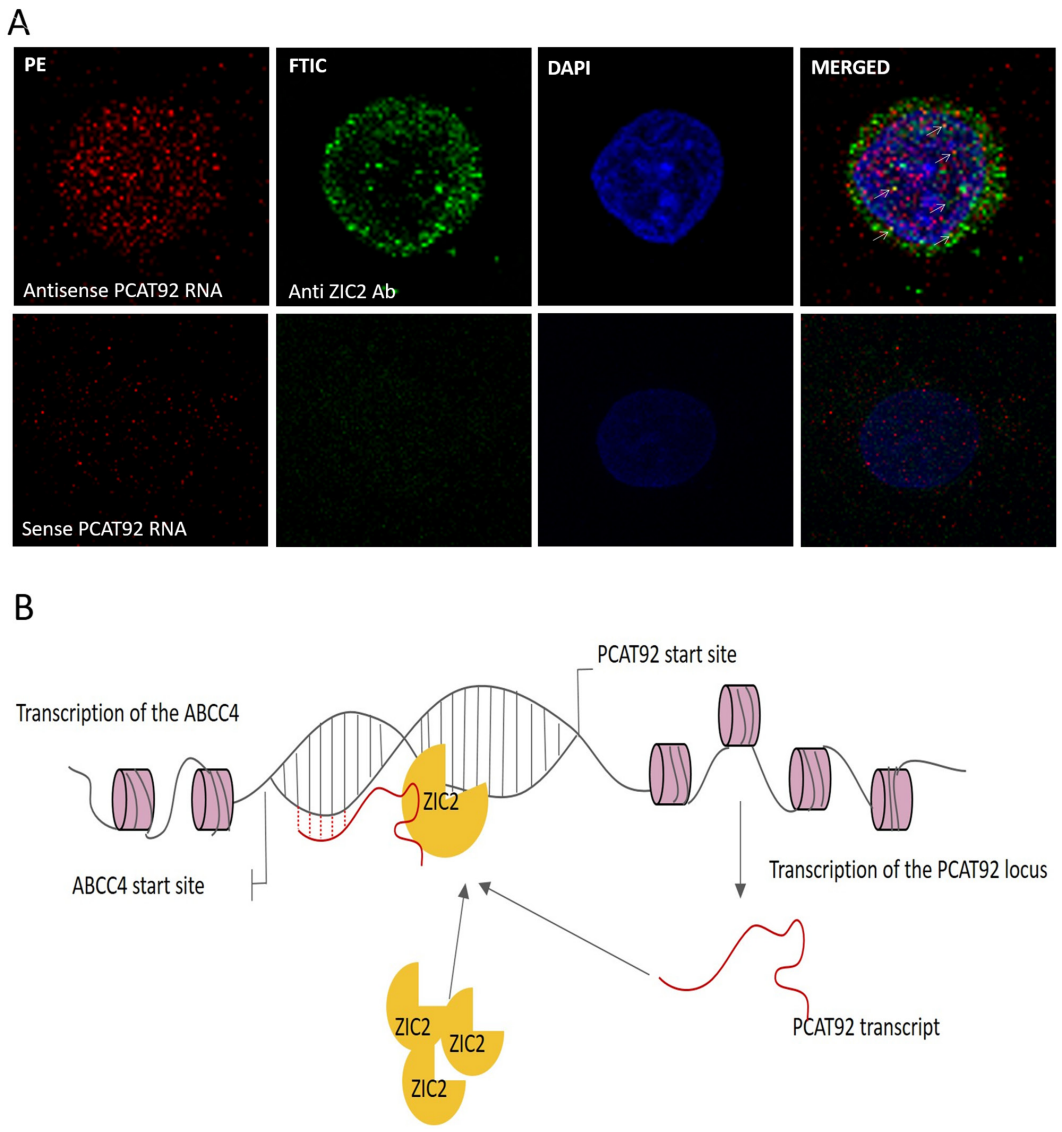
Cellular localization of PCAT92 and ZIC2 and hypothesis for the mechanism of PCAT92 involvement in PC **(A)** Co-localization of PCAT92 RNA and ZIC2 protein to the nucleus and cytoplasm has been shown. The yellow color in the nucleus (blue) indicates that both PCAT92 RNA and ZIC2 protein are interacting in the nucleus of the LNCaP cells. **(B)** Hypothesis for how PCAT92 might be regulating ABCC4 via protein ZIC2.

For PCAT92 to bind to chromosomal DNA the transcript need to be localized in the nucleus and if it helps in recruiting ZIC2 at ABCC4 promoter region, there should be an interaction among them in the nucleus of the cell. We performed *in situ* hybridization followed by immunofluorescence to study if both PCAT92 RNA and ZIC2 are co-localized in the nucleus of the cell. LNCaP cells were fixed in 4% paraformaldehyde. Sense and antisense probes of PCAT92 transcript was labelled with biotin. Fixed cells were permeabilized and hybridized with sense and anti-sense RNA probe. RNA probe was detected using streptavidin PE. Anti-ZIC2 antibody was added against ZIC2 and it was detected using streptavidin FTIC. DAPI was used as a counter stain to differentiate between nucleus and cytoplasm. As shown in Figure 7A antisense PCAT92 transcript gave red fluorescence in both cytoplasm and the nucleus of the cell indicating the presence of PCAT92 in both the compartment of the cell. Anti-ZIC2 anti body gave green fluorescence again in both cytoplasm and nucleus of the cell. Yellow fluorescence in the nucleus of the cell indicate co-localization of both PCAT92 and ZIC2 inside nucleus of the cell. The sense PCAT92 transcript was used as a negative control, which gave no signal within the nucleus of the cell. In both cytoplasm and nucleus co-localization was observed.

## DISCUSSION

In this paper we propose a novel mechanism by which a long non-coding RNA, PCAT92, drives cancer via regulation of a neighboring gene, ABCC4 on chromosome 13. Many lncRNAs and protein coding genes are known to be tightly co-expressed/co-regulated [33]. PCAT92 and ABCC4 falls under this category as they both were found to be co-expressed in multiple prostate cancer samples. PCAT92 knockdown in LNCaP cells inhibits both cell viability, cell proliferation and ABCC4 protein expression suggesting a role for PCAT92 in prostate cancer via the regulation of ABCC4. On the other hand, ABCC4 knockdown, while inhibiting cell viability and cell proliferation, has no effect on PCAT92 transcript expression suggesting that PCAT92 is upstream of ABCC4.

In order to understand the mechanism by which PCAT92 may be regulating ABCC4, transcription factor binding sites within the shared locus was examined. Among the significant transcription factors ZIC2 was also overexpressed in a cancer-specific fashion in all datasets studied here. ZIC2 overexpression have been reported in variety of cancer types including prostate [27], suggesting it to be an oncogenic transcription factor. ZIC2-GLI1 complex has already been shown to have oncogenic property [34]. Using pull down and EMSA we have confirmed ZIC2 binding to the ABCC4 promoter region. However under PCAT92 knockdown condition ZIC2 binding to the promoter region is diminished without any change in ZIC2 transcript level. This would suggest that PCAT92 may directly or indirectly play a role in recruiting ZIC2 to the ABCC4 locus. LncRNAs are known to interact with protein, for example MALAT1 has been shown to regulate alternative splicing by interacting with serine/arginine splicing factors and influencing their distribution in nuclear speckle domains [35]. Upon scanning the entire length of PCAT92 transcript for potential ZIC2 binding site using catRAPID tool a very significant binding motif was found at exon2-exon3 junction of PCAT92. Using Nano-ITC assay, we have validated ZIC2 interaction with the predicted junction of PCAT92 transcript with high significance compared to the antisense strand of PCAT92. To show *in vivo* interaction between PCAT92 transcript and ZIC2 protein we performed RNA immunoprecipitation. An enrichment in PCAT92 transcript under ZIC2 pull down condition further validated competitive interaction between PCAT92 transcript and ZIC2 within the cell. The hypothesis (Figure 7B) is that PCAT92 may be directly implicated in recruiting ZIC2 to the ABCC4 promoter region to regulate the expression of ABCC4.

One mechanism by which PCAT92 can stabilize the complex is by simultaneously interacting with chromosomal DNA. LncRNAs are known to form triplex with chromosomal DNA [32]. For example, lncRNA ANRASSF1 forms a RNA-DNA hybrid at the RASSF1A promoter and recruits the polycomb repressor complex PRC2, which in turns contributes to chromatin compaction [17]. Using Triplextator tool, it was shown that exon 3 of PCAT92 has the propensity to form triplex with the chromosomal DNA near the ABCC4 promoter region. We have built a model of the triplex and shown that it is energetically favorable. Using NanoITC we have validated interaction between the predicted region of PCAT92 transcript and the predicted chromosomal DNA near ABCC4 promoter region. In support of this hypothesis i) PCAT92-ZIC2 should be co-localized in the nucleus and ii) the two binding sites on PCAT92 and the two binding sites on the chromosome are far enough in the primary structure to avoid steric interference. Using co-localization studies, we have shown that PCAT92 and ZIC2 both are co-localized in the nucleus of the cell (Figure 7A). We observed presence of PCAT92 and ZIC2 in the cytoplasm of the cell as well. Currently we cannot comment on what can be the potential role of PCAT92 and ZIC2 in the cytoplasm of the cell, but we can strongly suggest that within the nucleus the complex is helping in the overexpression of ABCC4.

Interaction between the three molecules involved in the complex need not engage simultaneously to elicit the desired functional effect. However, even if one considers the complex to be stable over a short period, the binding sites within the same molecules are farther apart on the primary sequence to avoid steric hindrance. There are two parameters to consider while discussing the stoichiometry of the complex. i) Physical distance between the binding site of ZIC2 and chromosomal DNA on PCAT92 and ii) the promoter region and triplex forming region on the chromosome. On PCAT92 transcript, the ZIC2 binding motif and triplex forming regions are separated by more than 1000bps. On the side of the chromosome, ZIC2 promoter region (95957019-95957004) and the PCAT triplex forming site (95956510-95956500) are separated by roughly 500bps. The distances in the primary sequence are farther enough to avoid steric hindrance in the event the complex need to be stable over a short period to elicit the functional response.

Our work here have showcased that PCAT92 is upstream in the pathway which leads to the overexpression of ABCC4 in prostate cancer. This was further validated by performing RNA-Seq of two knockdown models of LNCaP cells. A large number of genes were commonly down-regulated under both the knockdown condition suggesting that they both are involved in the same pathway leading to prostate cancer. Pathway analysis of these commonly down-regulated genes enriched PI3K-Akt pathway. Crosstalk between PI3K-Akt pathway and Androgen Receptor pathway is well documented [29, 30]. Enrichment of PI3K-Akt pathway suggests one possible mechanism by which PCAT92 and ABCC4 are playing role in prostate cancer.

While lncRNAs, in general, are reported to have the potential to interact with diverse types of macromolecules both in the nucleus and cytoplasm, we believe that the sequence of interactions within the nucleus proposed here remains novel.

## MATERIALS AND METHODS

### Cell lines

Cell lines LNCaP, PC3, 22Rv1, RPWE-1 and HEK-293T were purchased from ATCC (ATCC, USA). LNCaP and 22Rv1 were maintained in RPMI media (Sigma-Aldrich, USA) supplemented with 10% FBS (Gibco, USA). PC3 was maintained in Ham’s F-12 media (Sigma-Aldrich, USA) supplemented with 10% FBS. RPWE-1 was grown in Keratinocyte Serum Free Medium (ATCC, USA) supplemented with bovine pituitary extract and human recombinant epidermal growth factor. HEK-293T was maintained in DMEM (Sigma-Aldrich, USA) supplemented with 10% FBS. All the cell lines were cultured in a humidified tissue culture chamber with 5% CO_2_ at 37°C.

### Generation of shRNA lentivirus particles

A CaCl_2_-DNA mix of 125μl was created containing 5μg each of pCMV-VSV-G, pCMV-Rev and pZIP-mCMV containing shRNA against the transcript (Transomic technologies, USA) and 12.5μl of 2.5M CaCl_2_. This mix was added drop-by-drop to 125μl of 2X HBSS while mixing constantly. HEK-293T was seeded at 1 × 10^6^ cells in a 35mm dish containing 2ml of DMEM supplemented with 10% FBS for 24 hours. Media was changed 1 hour prior to transfection. The transfection mix was incubated for 2 minutes at room temperature and added to the plate drop-by-drop. Eight hours post transfection, the media was replaced with 2ml of fresh DMEM supplemented with 10% FBS. The supernatant having viral particles was collected after 48 hours. GFP expression was used as a positive marker for the transfection.

### Transfection into LNCaP cells

LNCaP was seeded at 2 × 10^6^ cells in a 35mm dish with 2ml of RPMI supplemented with 10% FBS 48 hours before transduction. Freshly collected supernatant from transfected HEK-293T was centrifuged and added to plates. To ensure efficient viral uptake, 8 μg/ml of polybrene was added. The media was replaced 48 hours after transfection. The transfected cells were harvested and used for RNA and protein isolation.

### RNA extraction and qRT-PCR

Total RNA was isolated from cell lines using Trizol reagent (SRL, India). Total RNA was reverse-transcribed to cDNA using M-MuLV Reverse Transcriptase (NEB, USA) and random primer (NEB, USA). qRT-PCR was performed using a SYBR Green Realtime PCR Master Mix Kit (KAPA Biosystems, USA) in a StepOnePlus™ Real-Time PCR System (Applied Biosystems, USA). Beta-actin was used as a housekeeping gene. Relative fold change in gene expression were calculated using 2-ΔΔCt method, normalized with respective controls.

### Cell viability assay

Cell proliferation was evaluated using MTT assay. Transfected LNCaP wasand RPWE-1 cells with ABCC4, PCAT92 and scr shRNA were plated into 96-well plates (5 × 10^3^ cells/well). MTT assay was performed at an interval of 48 hours from Day 0 to Day 6. 10 μl MTT (5 mg/ml, SRL, India) was added and was incubated for 2 hours. The reaction was stopped by the addition of 100 μL DMSO. The optical density was measured at 570 nm using a microplate reader (TECAN, Switzerland). Each experiment was performed in triplicate.

### Cell proliferation and cell cycle analysis

Cell cycle status following the knockdown of both PCAT92 and ABCC4 was determined via flow cytometry analysis. Cells were harvested after knockdown and fixed in 80% ethanol at -20°C overnight. Following washing with PBS, cells were treated with RNaseA and then were stained with 0.1% (m/v) propidium iodide in PBST. Fluorescence was measured with a Gallios (Becton Dickinson). Each experiment was performed in triplicate.

### Western blot analysis

Total protein was extracted using RIPA buffer and quantified using Bradford’s’ reagent. Protein was loaded onto 10% SDS-PAGE, electrophoresed at 110 V for 2 hours and subsequently transferred onto PVDF membranes (BIO-RAD, USA). The membranes were incubated with mAb #12857, and mAb #4970 (Cell Signalling Technologies, USA) at a dilution of 1:1000 followed by incubation with mAb #7074 (Cell Signalling Technologies, USA) at a dilution of 1:10000. The blot was exposed to chemiluminescent reagent (BIO-RAD, USA) for visualization.

### RNA Seq library preparation and analysis

Sequencing libraries were prepared by using Illumina TruSeq RNA Library Prep Kit v2. The standard protocol recommended by the company was followed for library preparation. The quality and molarity of the libraries was evaluated by TapeStation (Agilent), and the samples were sequenced on the Illumina HiSeq2500, paired-read 100 bp. All analyses were carried out using the Tophat-Cufflinks pipeline [36, 37] with the following versions: Tophat v2.0.11, Bowtie2 v2.2.2.0, and Cufflinks v2.2.1. To align the RNA-seq reads to the genome, we first generated a Bowtie2 index using human reference genome (hg19) downloaded from UCSC genome browser and then run Tophat with default options. Transcript abundance (FPKM) and identification of differentially expressed genes was performed using Cuffdiff with default parameters. The differential expressed genes were filtered based on pvalue < 0.05 and log2 fold change of |1.5|.

### Co-localization studies

For co-localization analysis, the sense and antisense PCAT92 RNA probe was made. The DNA sequence corresponding to the RNA was cloned. Using T3 RNA polymerase (Thermo Scientific, USA) and Biotin-16-UTP (Roche, USA) both sense and antisense biotinylated probes were synthesized. LNCaP cells were fixed in 4% paraformaldehyde (Sigma Aldrich) at 4°C overnight. After washing the cells with 1X PBS, the cells were permeabilized using 0.1% Triton-X 100 (Sigma Aldrich, USA) and fixed again using 4% paraformaldehyde. Cells were washed and incubated with hybridization buffer for 2 hours at 37°C. Following this the biotinylated sense and antisense probe was denatured at 85°C for 5 minutes and added onto the cells and kept for hybridization at 37°C overnight in a humidified chamber. After washing, the free floating RNA were removed with RNaseA at 30°C for 30 minutes. The cells were washed and incubated with 1:10000 streptavidin-PE and mouse anti-ZIC2 antibody (sc517055; Santa Cruz Biotechnology), at room temperature for 1 hour. Cells were washed thrice with PBS and incubated with 1:200 anti-mouse biotinylated antibody (Sigma, USA) for 1 hour at room temperature. After washing with PBS the cells were incubated with 1:10000 streptavidin-FITC for 1 hour at room temperature. Cells were again washed thrice with 1X PBS and mounted using DAPI-DAPCO mix. The cells were observed under confocal microscope.

### Transcription factor binding site prediction

The common 5’ UTR between ABCC4 and PCAT92 loci (4305 bp) was given to MEME [38] for motif elicitation using palindromic as a parameter. Both the positive and the negative strands were used and the top three motifs were submitted to TomTom [39]. The precise binding locations of the transcription factors were determined using tools OProf and FindM [40]. The common 5’ UTR region was extracted from Eukaryotic promoter database (-4000 bp to +100 bp).

### ChIP RTPCR

Chromatin immunoprecipitation (ChIP) assays were performed using a SimpleChIP Enzymatic Chromatin IP kit (no. 9003; Cell Signaling Technologies). The standard protocol mentioned in the kit was followed. Briefly, four 100mm culture dishes containing 4×10^6^ LNCaP cells were grown and then cross-linked with 37% formaldehyde at a final concentration of 1% at room temperature for 10 minutes. Fragmented chromatin was treated with nuclease and subjected to sonication. Chromatin immunoprecipitation was performed with mouse anti-ZIC2 antibody (sc517055; Santa Cruz Biotechnology), rabbit anti-histone H3 (a technical positive control) (catalog no. 4620; Cell Signaling Technologies), and normal rabbit IgG (a negative control) (catalog no. 2729; Cell Signaling Technologies). After reverse cross-linking and DNA purification, immunoprecipitated DNA was quantified by real-time PCR SYBR Green Realtime PCR Master Mix Kit (KAPA Biosystems, USA) with primers for ZIC2 binding sites on the ABCC4 promoter and RPL30 exon 3 (catalog no. 7014; Cell Signaling Technologies). The enrichment of the desired region was calculated using qPCR. A serial dilution of the 2% input chromatin DNA (undiluted, 1:5, 1:25, 1:125) to create a standard curve and determine the efficiency of amplification. Standard curve was made for both positive control (RPL30 exon 3) and our test (ABCC4 promoter region). Using the two standard curve, the efficiency of amplification for both the regions was determined under all three the pull down condition. The entire experiment was repeated thrice for statistical significance.

### RNA immunoprecipitation

RIP is an antibody-based technique used to map *in vivo* RNA-protein interactions. The RNA binding protein (RBP) of interest is immunoprecipitated together with its associated RNA for identification of bound transcripts (mRNAs, non-coding RNAs or viral RNAs). Transcripts are detected by real-time PCR, microarrays or sequencing. Four 100mm culture dishes containing 4×10^6^ LNCaP cells were grown and then cross-linked with 37% formaldehyde at a final concentration of 1% at room temperature for 10 minutes. Following this 125mM of glycine was added to the media and incubated for 5 minutes at room temperature with gentle mixing. Cells were washed using ice-cold 1X PBS and were collected in a 15ml tube using scraper. Cells were pelleted at 2,500 g for 15 minutes and supernatant was discarded. Cells were resuspended in nuclear isolation buffer and were incubated for 20 minutes on ice with frequent mixing. Cells were centrifuged at 2,500g for 15 minutes and was resuspended in RIP buffer. Resuspended pellet was then split into two tubes for mock and IP. Chromatin was sheered using sonication followed by centrifugation at high speed to pellet down nuclear membrane and other debris. 2% of the lysate was aliquot and stored at -80°C until further use. To the remaining lysate, 10μg of mouse anti-ZIC2 antibody (sc517055; Santa Cruz Biotechnology) was added and incubated overnight at 4°C with gentle rotation. Post incubation, protein A/G beads were added and incubated for 2 hours at 4°C. Beads were pelleted by centrifuging at 2500 g for 1 minutes and was resuspended in RIP buffer. This step was repeated three times. Coprecipitated RNAs and RNA from 2% lysate aliquot was isolated by resuspending beads in TRIzol reagent (SRL, India). Total RNA was reverse-transcribed to cDNA using M-MuLV Reverse Transcriptase (NEB, USA) and random primer (NEB, USA). Following cDNA preparation, qPCR was carried out using primers specific to PCAT92 transcript. Primers specific to HEIH was also designed which was used as a positive control to validate protein-RNA binding. The entire experiment was repeated thrice for statistical significance.

### Evaluating lncRNA-protein interactions

The RNA-protein interactions based on sequence analysis was evaluated using catRAPID [41] with default parameters. The option ‘strength’ was used to compute the interaction strength between RNA-protein pair. The option ‘fragments’ was used to identify the residues interacting between the RNA-protein pair.

### Electrophoretic mobility shift assay

Double DNA sequences containing ZIC2 binding motif were synthesized. The single-stranded DNA oligonucleotides were annealed with their complementary strand and was 5’ end labelled with [γ-32P] ATP using T4 Polynucleotide Kinase (NEB, USA). Zinc finger domains of ZIC2 protein were PCR amplified and cloned in pET28a expression vector. The clone was sequenced verified and the induced using 0.5mM IPTG, at 16°C overnight. Induced protein was purified using His Tag affinity chromatography and eluted with the buffer containing 200 mM imidazole. 2μg of ZIC2-HIS protein and 100pmol of radiolabeled target dsDNA, was mixed in the binding buffer. The reaction solutions were incubated for 30 minutes at 37°C and subject to electrophoresis on 6% polyacrylamide gels prepared in 1×TBE (Tris-borate-EDTA) buffer for 16 to 18 hours at 100V at 4°C. Imaging was done using phospho imager (Kodak, NY, USA).

### Triplex formation and model building

To find all putative regions within PCAT92 transcript with triplex forming potential against the 4kb shared 5’UTR region on the chromosome, Triplexator computational pipeline was used [42] with all default parameters except for the minimum length of the triplex forming oligonucleotide. Lengths between 10 and 30 nucleotides was tried. The input file includes a FASTA format of ssRNA and double stranded chromosomal DNA to identify triplex forming regions within the input DNA.

A parallel RNA.DNA.DNA (R^*^DD) triple helical structure comprising U^*^AT and C^*^GC (Sequence 1) base triplets is generated conforming to a 12-fold helix [43] with a twist of 30 and rise of 3.27 Å. Stereochemistry of the third strand comprising C3’ endo nucleotide repeats was regularized by constrained-restrained molecular geometry optimisation and van der Waals energy minimization using X-PLOR [Brunger AT. X-PLOR Ver 3.851: Yale University, New York. 1996]. The generated model was further subjected to steepest descent energy minimization using the Sander module of AMBER 16.0 [D.A. Case, D.S. Cerutti, T.E. Cheatham, III, T.A. Darden, R.E. Duke, T.J. Giese, H. Gohlke, A.W. Goetz et.al (2017), AMBER 2017, University of California, San Francisco].

### Isothermal titration calorimetry

The isothermal titration calorimetry (ITC) experiments were performed at 25°C using Nano ITC (TA instruments, New Castle, DE). The protein sample was prepared as explained in Electrophoretic Mobility Shift Assay study. The predicted region of PCAT92 was cloned in pBS-SK plasmid followed by synthesis of sense and antisense RNA from the clone using T3 RNA polymerase (Thermo Scientific, USA) and T7 RNA polymerase (NEB, USA). 50μl of each sense RNA (60μM) and antisense RNA (60μM) was titrated into 300μl protein solution (5μM) with a fixed stirring speed of 300 rpm at 25 °C. Both RNA and Protein were dialysed in the same buffer (300 mM NaCl, 50 mM potassium phosphate, pH 8.0). Similarly to under the interaction between the PCAT92 RNA and ABCC4 DNA we performed ITC for the predicted PCAT92 and ABCC4 region. The oligos corresponding to both were obtained. 50μl of each PCAT92 RNA (10μM) and random RNA (10μM) was titrated into 300μl ABCC4 DNA solution (5μM) with a fixed stirring speed of 300 rpm at 25 °C. Both RNA and DNA were dialysed in the same buffer (10mM Sodium Acetate, 0.2M Sodium Chloride, 20mM Magnesium Chloride, pH4.5). For both the titration experiment, 5μL of RNA was dropped for 10 injections. To achieve complete equilibration, the spacing time between each injection was set to 300 seconds. All measurements were repeated at least twice. Using Nano ITC NanoAnalyze software, the raw data was integrated, corrected for nonspecific heats and analysed according to independent model.
